# Biomechanical microenvironment in peripheral nerve regeneration: from pathophysiological understanding to tissue engineering development

**DOI:** 10.7150/thno.74571

**Published:** 2022-06-27

**Authors:** Lingchi Kong, Xin Gao, Yun Qian, Wei Sun, Zhengwei You, Cunyi Fan

**Affiliations:** 1 Department of Orthopedics, Shanghai Jiao Tong University Affiliated Sixth People's Hospital, Shanghai, 200233, China.; 2 Shanghai Engineering Research Center for Orthopaedic Material Innovation and Tissue Regeneration, Shanghai, 200233, China.; 3 Youth Science and Technology Innovation Studio of Shanghai Jiao Tong University School of Medicine, Shanghai, 200233, China.; 4 State Key Laboratory for Modification of Chemical Fibers and Polymer Materials, Shanghai Belt and Road Joint Laboratory of Advanced Fiber and Low-dimension Materials, College of Materials Science and Engineering, Shanghai Engineering Research Center of Nano-Biomaterials and Regenerative Medicine, Donghua University, Shanghai, 201620, China.

**Keywords:** Peripheral nerve injury, Peripheral nerve regeneration, Biomechanical phenomena, Mechanotransduction, Biomaterials, Tissue engineering

## Abstract

Peripheral nerve injury (PNI) caused by trauma, chronic disease and other factors may lead to partial or complete loss of sensory, motor and autonomic functions, as well as neuropathic pain. Biological activities are always accompanied by mechanical stimulation, and biomechanical microenvironmental homeostasis plays a complicated role in tissue repair and regeneration. Recent studies have focused on the effects of biomechanical microenvironment on peripheral nervous system development and function maintenance, as well as neural regrowth following PNI. For example, biomechanical factors-induced cluster gene expression changes contribute to formation of peripheral nerve structure and maintenance of physiological function. In addition, extracellular matrix and cell responses to biomechanical microenvironment alterations after PNI directly trigger a series of cascades for the well-organized peripheral nerve regeneration (PNR) process, where cell adhesion molecules, cytoskeletons and mechanically gated ion channels serve as mechanosensitive units, mechanical effector including focal adhesion kinase (FAK) and yes-associated protein (YAP)/transcriptional coactivator with PDZ-binding motif (TAZ) as mechanotransduction elements. With the rapid development of tissue engineering techniques, a substantial number of PNR strategies such as aligned nerve guidance conduits, three-dimensional topological designs and piezoelectric scaffolds emerge expected to improve the neural biomechanical microenvironment in case of PNI. These tissue engineering nerve grafts display optimized mechanical properties and outstanding mechanomodulatory effects, but a few bottlenecks restrict their application scenes. In this review, the current understanding in biomechanical microenvironment homeostasis associated with peripheral nerve function and PNR is integrated, where we proposed the importance of balances of mechanosensitive elements, cytoskeletal structures, mechanotransduction cascades, and extracellular matrix components; a wide variety of promising tissue engineering strategies based on biomechanical modulation are introduced with some suggestions and prospects for future directions.

## Introduction

Peripheral nerve injury (PNI) is a common and challenging issue in clinical practice due to irreversible nerve tissue damage and difficult repair 1, 2. A wide variety of factors including high energy trauma, chronic disease and surgical complication will lead to an uncontrollable pathophysiological process and subsequently result in sensory and/or motor disability and poor life quality of patients 3, 4. At the aspect of microstructure, the peripheral nerve is composed of neurons, myelin sheaths and connective tissues (e.g. epineurium, perineurium, endoneurium, vessels and immune cells). PNI could be divided into three different severity level conditions according to the Seddon classification: neurapraxia, axonotmesis and neurotmesis 5. Neurapraxia is defined as a major electric conducting blockage due to myelin damage without axonal structural injury and Wallerian degeneration. Axonotmesis refers to complete axonal disconnection while the myelin remains intact. Neurotmesis, the most severe type of PNI, is characterized by the physical transection of neurons, myelin sheaths and other supporting tissues. Currently, the most ideal clinical outcomes can be achieved by simple end-to-end sutures for tension-free nerve gaps, while autograft remains the golden criteria for critical defects (greater than 5 mm in adults) in the clinical application 6, 7. Although autograft almost meets the peripheral nerve repair criteria, such as high biocompatibility, natural tissue composition and appropriate extracellular matrix (ECM) support, the shortages that limited donor availability, secondary damage, size mismatch and neuroma formation cannot be ignored 1. Consequently, it calls for an alternate strategy that combines both tissue accessibility and biomimetic qualities to alleviate the therapeutic dilemma.

**Figure 1:**
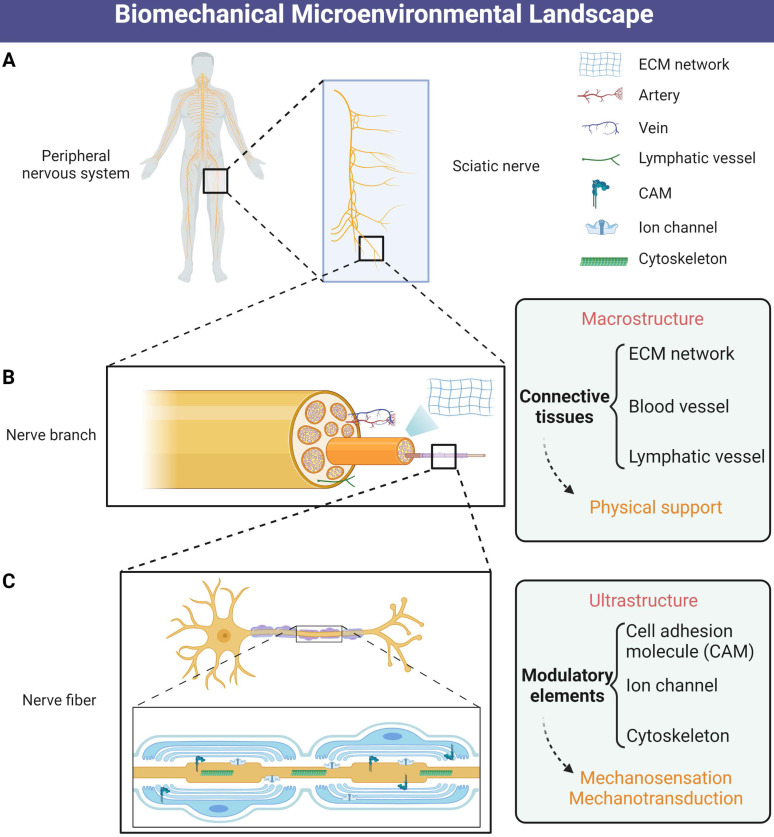
**Schematic illustration of biomechanical microenvironmental constitutions of peripheral nerve. (A)** Distribution of the human peripheral nervous system. **(B)** The macrostructure of physical support consists of ECM networks and vascular tissues. **(C)** The ultrastructure of biomechanical microenvironment includes mechanosensitive and mechanotransductive elements including CAMs, mechanosensitive ion channels and cytoskeletons. Abbreviations: ECM, extracellular matrix; CAMs, cell adhesion molecules.

The microenvironment of peripheral nerve regeneration (PNR) is a dynamic landscape of both chemical and biomechanical cues that regulate tissue regrowth and cell behaviors including proliferation, migration, differentiation, maturation and paracrine pattern 3, 8, 9. Compared to chemical signals, the role of mechanical stimulation is less explored but important for nerve system development and function maintenance 10, as well as the process of PNR following injury 11, 12. Physical supports from peripheral connective tissues and ECM protect functionalized neurons from external detrimental factors. In addition, the biomechanical microenvironment means more local and systematic biomechanical conditions including ECM stiffness, mechanosensitive element status and mechanotransduction signal received by intracellular cytoskeleton and membrane receptors than external physical stimulation 13 (Figure 1). Electrical signal conduction of both neuron and Schwann cell myelination is regulated by mechanotransduction molecules 12, 14, such as RhoA, yes-associated protein (YAP) and transcriptional coactivator with PDZ-binding motif (TAZ), indicating that appropriate biomechanical microenvironment is necessarily required for PNR.

The past decades witnessed the fast development of tissue engineering techniques that have enriched the therapeutic strategies for PNI 15, 16. A range of nerve grafts and nerve guidance conduits (NGCs) fabricated by natural or synthetic substances have been designed and applied in the PNI model, part of which managed to achieve relatively ideal outcomes comparable to autograft 17, 18. The material principle, scaffold design and specific modification enable these biomimetic grafts to optimize mechanical properties including structural characterization, stiffness and hydrophilicity 15. As for biomechanical microenvironment simulation, a large number of longitudinally oriented scaffolds have been manufactured utilizing various techniques 19, 20. Piezoelectric materials by virtue of their capacity of transforming mechanical stimulation into electrical signal and the nature of axonal electric conduction are also employed to repair peripheral nerve defects 18, 21. Of note, several topological designs displaying porous and aligned architectures, internal multi-channels, and patterned surfaces tended to mimic natural biomechanical microenvironment for improving cell migration and attachment 22. Therefore, those of biomimetic grafts and NGC, piezoelectric scaffolds or topological designs serve as potential PNR strategies are promising through biomechanical adaptation and modulation.

In this review, we will systematically discuss the effects of the biomechanical microenvironment on peripheral nervous system development and function maintenance to inspire in-depth investigation, as well as complicated responses of peripheral nerve tissues, cell populations and molecules to PNI. Furthermore, the applications and limitations of those biomaterials and tissue engineering strategies by modulating biomechanical microenvironment for PNI management will be also summarized, which provides basic principles for preclinical research and even clinical translation.

## Histological development and function maintenance of peripheral nervous system in the biomechanical microenvironment

**Figure 2:**
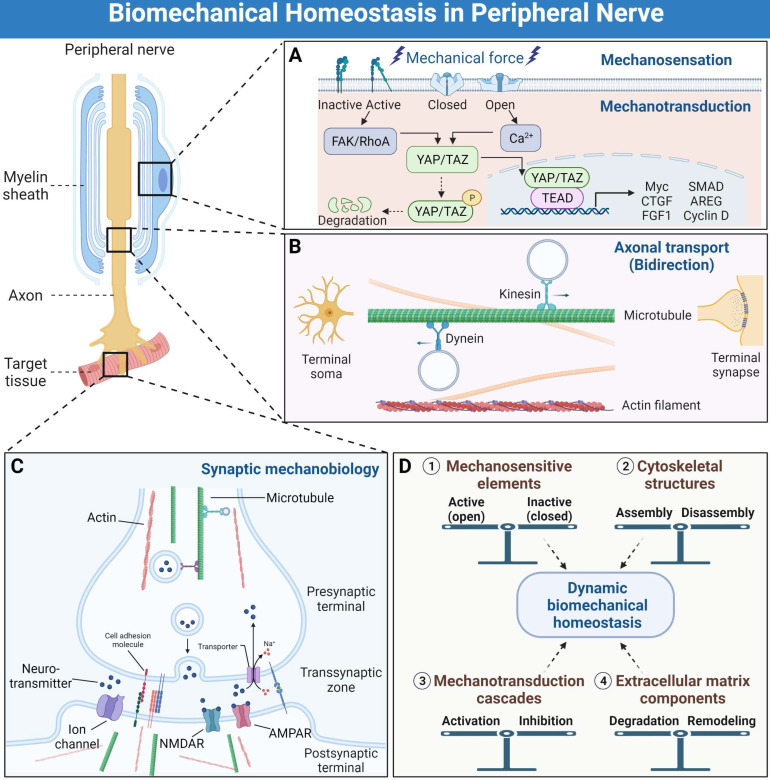
**Biomechanical mechanisms of peripheral nervous system development and function maintenance. (A)** The simplified processes of mechanosensation and mechanotransduction in peripheral nerve system. **(B)** The bidirectional axonal transports by means of microtubules and molecular motors, as well as other cytoskeletal types. **(C)** Synaptic mechanobiology of peripheral nerve. **(D)** The dynamic biomechanical homeostasis is maintained by balances of mechanosensitive elements, cytoskeletal structures, mechanotransduction cascades and extracellular matrix components.

Biomechanical microenvironment always determines life formation and activities; therefore, cellular guidance and assembly into specialized tissues under mechanical strain are essential processes for multicellular life 23. Continuous biomechanical signals, such as the Hippo pathway activation, are required for histological development and function maintenance of peripheral nervous system 24, 25. A series of genes have been identified in the peripheral nervous system being directly regulated by mechanical stimuli, especially during the embryonic development period 26, 27. Various cell types also displayed differential biomechanical microenvironment preference and cellular biological behavior in response to mechanical stimuli 11, 28. These well-organized interactional networks and transduction cascades contribute to the establishment of biomechanical microenvironmental homeostasis. We propose that the homeostasis is dynamically maintained by balances of (1) mechanosensitive elements, (2) cytoskeletal structures, (3) mechanotransduction cascades, and (4) extracellular matrix components (Figure 2).

### Neuron

The occurrence and outgrowth of neurons always depend on interaction with extracellular components in the dynamic microenvironment, intercellular communications as well 28-30. Mechanical properties of the extracellular substrate modulate signal transduction for biological activities of the axons, such as electrical conduction and protein transport 31, 32. Stiffness-dependent axon outgrowth requires actin-adhesion coupling mediated by laminin and ligand molecules on the substrate 11, 29, 30, which may be triggered and modulated by the mechanical effectors (YAP/TAZ) of Hippo pathway induced laminin receptor expression as described by a recent study 24. Both endogenous and exogenous mechanical factors are necessary for the well-organized radial sorting of peripheral nerve axons.

Internal force transmission elements including cell adhesion molecules (CAMs), molecular motors (e.g. kinesin, myosin and dynein) and intracellular filamentous structures (e.g. actin and microtubules) play an important role in cell motility, adhesion and axonal transport, contributing to growth cone activities and axon extension 33-37. Growth cones at the tip of extending axons generate traction force for axon outgrowth by transmitting the force of actin filament retrograde flow originating from actomyosin contraction and F-actin polymerization, to adjacent cells or adhesive substrates through membrane clutch and CAMs 29, 31, 38. A cluster of mechanosensitive molecules between the actin filament flow and substrate govern dynamic signal transduction for neural activities 34, 35. For instance, shootin1 interacting with cortactin or L1 generates traction force for axon outgrowth and guidance, while impairing these interactions, either by shootin1 RNA interference or disturbing the interaction between shootin1 and actin filament flow, will inhibit shootin1-dependent axon outgrowth 30, 38. Therefore, the actin retrograde flow-clutch protein-adhesive substrate axis is mainly responsible for endogenous mechanotransduction of neurons in a stiffness-dependent manner because substrate stiffness could directly modulate the interactional efficiency between clutch proteins and adhesion molecules 29.

In addition to endogenous mechanisms, external factors in the biomechanical microenvironment have also been identified to regulate neural activities in peripheral nervous system. As early as 1984, it was found that axons extended in response to stretch from microenvironment 39. Up to now, it has been well established that neurite growth and expansion are driven by not only biochemical but also mechanical signals 40, 41. Briefly, motor neurons prefer rigid substrates similar to the elasticity of muscle, while forebrain neurons prefer softer conditions that approximate brain tissue elasticity, whereas RhoA balances contractile and adhesive forces in response to substrate elasticity 11. Axonal stretch under the complicated biomechanical microenvironment, in turn, has been identified as a regulator for axonal transport, and different levels of stretch could physiologically or pathologically modulate neural behaviors during stress reaction or traumatic axonal injury 42, 43.

### Synapse

Neural synapse, the intercellular asymmetrical junction, is in charge of transmitting biochemical and biomechanical information between axons and target cells. Synapses were thought to be static structures for a long time. Nevertheless, they are now considered as dynamically changing units that consist of presynaptic active zone, postsynaptic terminal and transsynaptic bond 44. From the perspective of mechanical cue-induced synapse formation, the following four steps are necessary: the elongation of neurites, structural attachments between neurites and their targets, mechanical force-mediated survival of the axonal branch and complete synapse formation 45. Generally, the elements of synaptic cell adhesion molecules (sCAMs), cytoskeletal filaments and mechanosensitive ion channels altogether contribute to biomechanical microenvironment homeostasis and function 46-48. A number of sCAMs such as cadherin, integrin and ephrin are found in synapses, part of which form transsynaptic bonds to align the presynaptic active zone with the postsynaptic density. For instance, N-cadherin-mediated extracellular neuron-neuron interactions are indispensable for maintaining neurite outgrowth and branching, as well as synapse formation and stabilization 49, 50. It is established that a filamentous actin network with complex spatiotemporal behavior controls the dendritic morphology, which is thought to be crucial for activity-dependent synapse plasticity together with actin-binding proteins 51. In addition, it is known that the external or internal forces are sufficient to induce conformational changes on the gating domain of an ion channel to modulate ion transportation and convert mechanical stimuli into electrical or biochemical signals, largely contributing to synaptic communication function 52. Moreover, mechanical signals mediated by both cytoskeleton and sCAMs could also drive mechanosensitive ion channel activation through multiple binding partners of the adaptor proteins and assembly of channels 53, 54. Several central nervous disorders have been identified as results of dysfunction of these elements 55, despite the fewer relations of PNI to the synaptic mechanical microenvironment. Therefore, synaptic mechanical structure and signal transduction altogether maintain a normal neural network.

### Schwann cell

As is well known, Schwann cells in peripheral nervous system play key roles in insulating between nerve fibers and protecting from harmful stimuli by forming myelin sheaths around axons, which also improves electrically transduction efficiencies of action potentials 56. The exposure of Schwann cells to mechanical cues depends on the stiffness and elasticity of peripheral nerves that are partially determined by the connective tissue and ECM surrounding the nerve fibers 56, 57. Therefore, the biomechanical microenvironment of Schwann cells includes mechanical properties of entire nerve fibers and external mechanical forces. Most of the Schwann cell myelin segments often consist of compact myelin, while compact myelin is interrupted by regions of uncompact part called Schmidt-Lanterman incisures in charge of intracellular transport 58, 59. In addition to their morphology, Schwann cells also contribute to the mechanical resistance of peripheral nerves by secreting the basal lamina, an essential component wrapping axon-myelin sheath unit for Schwann cell development and biomechanical microenvironment maintenance 24, 60, 61.

Extracellular and intracellular mechanotransduction pathway regulation always dynamically maintains the cell shape of Schwann cells, as well as cell proliferation, differentiation and maturation. Those membrane molecules on the cellular surfaces such as integrins are initially sensitive to mechanical signals, and subsequently activate a signaling cascade involving FAK, Src, PI3K and JNK pathways, or the formation of actomyosin filaments 62-65. YAP and TAZ, two transcriptional activators in the Hippo pathway as downstream of mechanosensitive elements, were dephosphorylated and activated through the Hippo pathway in SCs by Crb proteins 66. Thereafter, YAP and TAZ are translocated to the nucleus to regulate gene expression 67. In addition to regulating YAP and TAZ, the F-/G-actin ratio also participates in regulating the translocation of myocardin-related transcription factors (MRTFs) from the cytoplasm into the nucleus to activate serum response factor (SRF)-dependent transcription 67, 68. Indeed, MRTFs not only regulate YAP/TAZ response but also directly modulate transcriptional targets 69. Developing Schwann cells require YAP/TAZ to enter S-phase before proliferation, without which it will fail to generate sufficient Schwann cells for axon sorting in order 25, 70. As for differentiation and maturation, Schwann cells require YAP/TAZ to upregulate Krox20 for myelination 25. Moreover, nuclear YAP/TAZ is selectively expressed in myelinating Schwann cells, regulating both developmental and adult myelination by driving TEAD1 to activate Krox20 25, 56. However, a recent study pointed out that short or long-term myelin maintenance was not impaired by defect in YAP and TAZ 70, and interestingly another research indicated that TAZ was more required in Schwann cells for radial sorting and myelination and that YAP was redundant when TAZ exists 24. Further work will be carried out to address the contradictory issue. Of note, despite the wide involvement of YAP/TAZ in above activities, whether they are associated or not with mechanical stimuli remains unclear.

Mechanosensitive ion channels governing mechanosensation and transduction have also attracted wide attention in the area of neuroscience. The past decades have witnessed some progress in identification of novel ion channels expressed in nearly all cell types by means of probing techniques, including mechanosensitive potassium channels and Piezo ion channels 71-73. As a type of Ca^2+^-permeable non-selective cation channel, Piezo2 is more associated with the nervous system due to its role in sensory nerve fiber, including sensing touch and lung stretch 74, while Piezo1 channel was shown to respond to different micropillar properties, including greater stimulation with increased pillar spacing and reduced stiffness 75. Although recent studies showed that Piezo activation tended to inhibit axonal regeneration 76, related knowledge gaps need substantial work to close for clarifying mechanisms. Additional mechanosensitive channels known to participate in the nervous system include a subset of the transient receptor potential (TRP) family of proteins, N-type channels, N-methyl-D-aspartate (NMDA) receptor and TWIK-related arachidonic acid-activated K^+^ (TRAAK) channels, all of which not only allow ion influxes and thus enable intercellular communication, but also respond to biomechanical microenvironment for downstream signaling to help decision-making in the life cycle of Schwann cells 77-79.

## Biomechanics-based pathophysiological responses and regenerative processes after PNI

**Figure 3:**
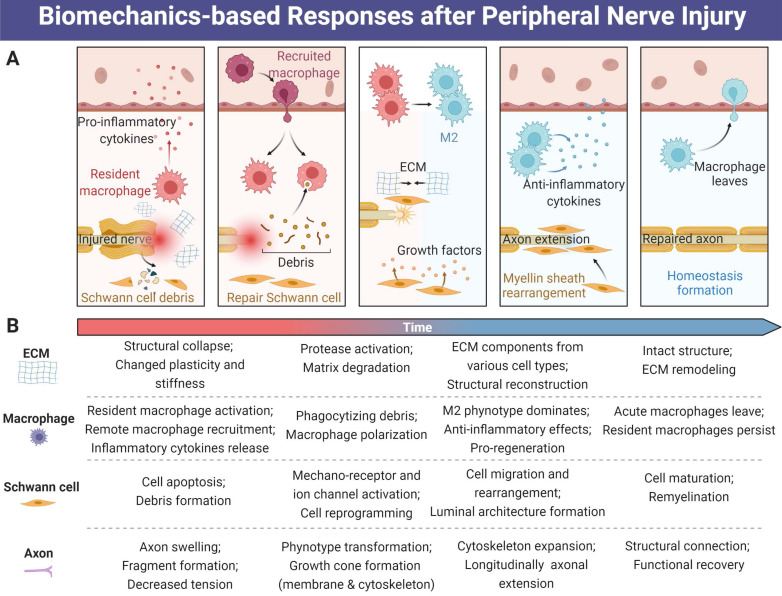
**Biomechanical factors-induced responses for tissue repair and functional recovery after peripheral nerve injury.** (A) Extracellular matrix, macrophages, Schwann cells and axons are always modulated by the biomechanical microenvironment during PNR. (B) The time-dependent activities among ECM, macrophage, Schwann cell and axon during PNR. Abbreviations: ECM, extracellular matrix; PNR: peripheral nerve regeneration.

After axotomy, neural tissue shifts from a functional transmitter to a regenerative phenotype, activating a series of molecular pathways that drive neural survival and axonal repair 8, 80. PNI induces a cascade of events, at the molecular, cellular, and system levels, initiated by the biomechanical microenvironment changes at the suffering sites 13, 81 (Figure 3). Although several animal studies indicated that the mechanical property changes of peripheral nerve following compression, and the association of mechanical stretch with *in vivo* functional and histological outcomes 82, 83, deep exploration in animal models and humans is largely needed. Up to now, most distinct mechanism relevant to biomechanics in the area of PNR have been found *in vitro*, but it is insufficient to reflect *in vivo* conditions 84. Mechanical factor-related mechanisms involved in these changes contain varied ECM stiffness and plasticity, physical architecture-induced cell alignment, mechanosensitive ion channel activation or inactivation of nearly all cell types, cytoskeletal filament rearrangement, and reorganization of sensory and motor nerve fibers 11, 81, 85-87, all of which will be the potential therapeutic targets in the future.

### Extracellular substrate

Nervous ECM directly suffers the visible or undetectable damage in case of traumatic PNI, where its mechanical properties tend to be changed due to axonal interruption to a varying degree 80. Traumatic PNI, especially peripheral nerve defect is often accompanied by muscle and supportive connective tissue destruction, further destroying the inherent biomechanical microenvironment of peripheral nerve. On the other hand, biochemical changes such as protease activation and neuropeptide secretion in the local microenvironment could indirectly affect the plasticity and stiffness of extracellular components 81, 88. Taken together, a majority of mechanosensitive molecule activation and intracellular mechanotransduction are initiated by changed mechanical properties of ECM after acute PNI. For example, neural/mesenchymal stem cell homing and differentiation are prerequisites for neural reorganization at the cellular level, and these cell types are regulated by not only biochemical signals but also biomechanical microenvironment 13, 89. Stemness maintenance or differentiation of neural stem/progenitor cells are closely associated with nerve repair and regeneration, as Madl *et al*. 90 designed a three-dimensional (3D) neural progenitor cell-loaded hydrogel model and demonstrated that the degradability and remodeling of ECM positively correlated with neural progenitor cell stemness, which attributes to cadherin-mediated intercellular communication and β-catenin signaling activation without cytoskeletal tension generation. Therefore, the injury degree of peripheral nerve matrix may determine stem/progenitor cell homing and function, while the intrinsic matrix stiffness within the range from 0.5 kPa to 50 kPa less links to stem/progenitor cell fate decision 90, 91. Cell responses and structure reconstruction driven by changed biomechanics of ECM are described in detail below.

### Macrophage

A few immune cell types have been identified to participate in nerve regrowth after PNI, especially in the early stage, as reactive responses to acute inflammation and the requirement of new tissue regeneration. Although a wide range of chemokines released from injury sites as primary factors recruit inflammatory and immune cells and regulate their phenotypes, biomechanical microenvironment-induced cell behaviors are still indispensable 92. Recent findings showed the involvement of macrophages and their polarization to M2 phenotype enabled the migration, proliferation and remyelination of SCs, which subsequently facilitated axonal extension for better restoration of sciatic nerve function. During this process, the physical architecture impairment of nerve bundle trigger monocytes infiltration and transfer to macrophages; thereafter, macrophage tends to polarize to M2 phenotype on integral oriented basal matrix, while M1 phenotype on irregular basal matrix 19, 20, 93, consolidating the fact that support structure and biomechanical properties guide cell behaviors of macrophage. Accumulating evidence indicated mechanical modulation-induced YAP/TAZ activation may lead to M2 polarization of macrophages 94, but more details need to clarify.

In turn, macrophages are also identified to clear debris for subsequent neural regrowth, and this process exerts an effect on biomechanical microenvironment modulation. Briefly, resident macrophages accounting for 2-9 % of total cells in peripheral nerves are endowed with phagocytic abilities collaborating with Schwann cells and microvascular endothelial cells in the early stage of Wallerian degeneration, while recruited monocytes or macrophages play a major role in long-term microenvironmental regulation 95-97. Live-cell imaging study also demonstrated that macrophages assembled in the injury sites long before axon fragmentation, and axonal debris can be engulfed by axon fragmentation-triggered invasion of macrophages in a zebrafish model 98. Several M2-derived cytokines including Gas6 and proteases play a key role in the regulation of Schwann cell debris clearance, ECM remodeling and immature Schwann cell migration and maturation 99, 100. Unfortunately, the roles of other immune cell types in biomechanical modulation and crosstalk within macrophages are still obscure, as well as their spatial-temporal variation.

### Schwann cell and myelin sheath

Local mechanical support will be completely or incompletely destroyed when disruption or crush occurs, and the intact structure of myelin sheath and Schwann cell subsequently collapse. Simultaneously, inflammatory response and immune regulation will also be triggered to remodel the biomechanical and biochemical microenvironment 101-103. Both of them lead to myelin sheath debris clearance and renewed biomechanical microenvironment reconstruction, as well as remote Schwann cell migration, maturation and myelin sheath formation. Following traumatic nerve injury, Schwann cells in axotomized nerve rapidly dedifferentiate, reprogram and proliferate as they convert to regeneration promoting phenotype repair Schwann cells that are also identified as “activated status” 89, 104. During this process, the acquisition of mesenchymal traits and a Myc module are observed in Schwann cells, and the mature cell population subsequently guides axon regeneration 89, 105.

As for the related mechanisms, the PNI-induced CAMs activation on Schwann cell surface not only triggers intracellular downstream mechanotransduction but also is responsible for communications with neurons and ECM 106, 107. For instance, Ninjurin 1 plays a crucial role in the remyelination process of nerve/glial antigen 2^+^ cells including immature Schwann cells after PNI through accelerating Schwann cell maturation 106. Recent findings showed that increased TAZ levels may be a major compensatory factor for nerve injury, while YAP expression levels remain stable and ablation of YAP alone fails to affect peripheral nerve remyelination 108. These data hint toward a specific role of TAZ in peripheral nerve remyelination. However, independent work demonstrated that YAP alone plays a role in the modulation of internodal length and positively regulates remyelination and myelin elongation, which provides the necessary tracks for axon guidance and supports axonal regeneration 66, 104. Although YAP/TAZ participated in remyelination, there was no direct evidence that TAP/TAZ were activated by mechanical stress-induced cascades. As c-Jun, FAK and YAP/TAZ are commonly involved in tissue development and/or cell fate decision, the biomechanical microenvironment-modulated Schwann cell responses resemble some aspects of embryonic development and trigger the inherent neural regenerative potential, in line with the principle of tension stress-driven histogenesis 109, 110. In addition to cell fate modulation, paracrine patterns of Schwann cells are also affected by mechanical stimuli capable of regulating microRNA and gene expression levels in exosomes derived from Schwann cells 111.

### Axon

The fact that “ECM-Schwann cell-neuron axis”-driven axonal regrowth in biomechanical microenvironment has been well accepted 87, 112. Moreover, injured peripheral axons are also directly modulated by ECM stiffness and elasticity to adapt to the changed biomechanical microenvironment and restore their function 113, 114. Neurons after axotomy rapidly switch from a transmission state to a growth state employing mechanosensitive elements including CAMs, mechanical ion channels and cytoskeletons, with changes in the expression levels of genes that encode for transcription factors involved in cell survival and neurite outgrowth. Generally, Schwann cell and immune cell pave the way for axonal extension in the conditions of post-peripheral nerve defect regeneration, whereas luminal architecture formed by myelin sheath offers the most optimized supportive channel and biomechanical microenvironment 87. In turn, the tissues in charge of guiding neuronal extension are also dependent on axonal activities upon nerve injury. For example, axons regulate the transcriptional activity of YAP/TAZ in adult Schwann cells, which leads to differentiation and maturation of Schwann cells and remyelination; further work is worthy of developing to understand the axon-dependent mechanisms 12.

As mentioned above, the cytoskeleton plays a crucial role in the formation and maintenance of biomechanical homeostasis, and damaged nervous terminals outgrowth is also dependent on cytoskeleton rearrangement and activity after PNI through their mechanosensitive and mechanomodulatory characterizations 115, 116. Considering that axonal disruption always leads to cell swelling and retraction of dendrites when exposed to both detrimentally biomechanical and biochemical factors, the robust stability and organization of microtubules define the fate of lesioned axonal stumps to become advancing growth cones instead of nongrowing retraction bulbs 117, 118. Furthermore, a study showed that conditioned sciatic nerve injury failed to increase the somatic size of sensory neurons but promoted the appearance of longer and larger neurites and growth cones 8. An atomic force microscope was also employed to investigate changes of live neurons in morphology and membrane mechanical properties of conditioned neuronal somas following sciatic nerve injury, and the results showed neurons displaying a regenerative growth pattern became softer and more elastic than control ones although they showed similar shapes and sizes 8, 85. The increase of the growth cone membrane elasticity suggests a modification in the framework of the main structural proteins.

Traumatic PNI-induced neuropathic pain due to peripheral sensitization, in particular mechanical allodynia, is closely associated with abnormal activities of mechanosensitive receptors and ion channels 119-121. Fast-conducting myelinated high-threshold mechanoreceptors (AHTMR) thought to transmit acute nociception from the periphery were identified to contribute to threshold-related withdrawal behavior after nerve injury, indicating injured axonal mechanoreceptors regulated by biomechanical microenvironment could result in mechanical pain formation 122, 123. Spontaneous ectopic discharges derived from dorsal root ganglion neurons or injury sites are a key factor in the initiation of neuropathic pain, where axonal hyperpolarization-activated cyclic nucleotide-gated cation (HCN) channel accumulation enhances mechanical hypersensitivity and plays an important role in ectopic discharges from injured nerves, while specific HCN blocker successfully relieves the ectopic discharges from injured nerve fibers with no effect on impulse conduction 124.

## Biomechanics-based tissue engineering strategies for PNI management

Current surgical approaches and biochemical methods often fail to manage PNI, especially critical peripheral nerve defect, but fortunately, the rapid development of tissue engineering techniques has shed light on addressing this concern. In the past decades, several tissue engineering research directions have been confirmed based on biomechanical microenvironmental characteristics of PNR. Two-/three-dimensional topological NGCs and piezoelectric scaffolds emerge 15, 22, 125-127, which undoubtedly provides promising alternatives for severe PNI and peripheral nerve defect repair.

### Optimized mechanical properties

Tissue engineering scaffolds as nerve grafts applied in PNI management are expected to display optimized biomechanical properties for better neural rearrangement and functional recovery. The desirable mechanical properties of nerve grafts are expected to recreate a natural ECM-like physical structure and mechanical strength for neural repair 15, 128. A wide variety of natural or synthetic biomaterials including collagen, polycaprolactone (PCL) and poly-L-lactic acid (PLLA) have been widely fabricated into nerve grafts in peripheral nerve defect models 129-132. All raw materials selections, fabrication techniques, and environmental conditions contribute to elastic modulus, elongation at break and microstructure of nerve grafts. The uniform and optimized mechanical properties of those products usually vary within a certain range that can be used as a reference for related studies in the future (Table 1).

Typically, PCL approved by Food & Drug Administration (FDA) as a type of biomaterial with appropriate mechanical properties, perfect biocompatibility and satisfactory degradability, is one of the most popular basic chemicals in the field of PNR 17, 18, 133, 142, 143. Among previous studies, the elastic modulus of PCL scaffolds varies from 30 MPa to 80 MPa due to differential fabrication techniques. These studies indicated that adequate elastic modulus ensured better support for long-term *in vivo* experiments, since poor elastic modulus will render grafts to collapse under the local mechanical strain 15, 143. Extra supplements such as collagen and gold nanoparticles are also employed to improve elastic modulus and elongation at break of scaffolds 125, 142, 144. As mentioned above, composited nerve grafts as regenerative platforms during PNR aim to display agreeable stiffness for refined mechanical signal activation 78, 145. A wide range of specialized physical architectures of nerve grafts including net-like filament, porous structure and aligned micro-/nano-topography also contribute to the improvement of mechanical properties, affecting repair outcomes of neural fiber 22, 93, 146, 147.

### Two-dimensional (2D) aligned designs

**Figure 4:**
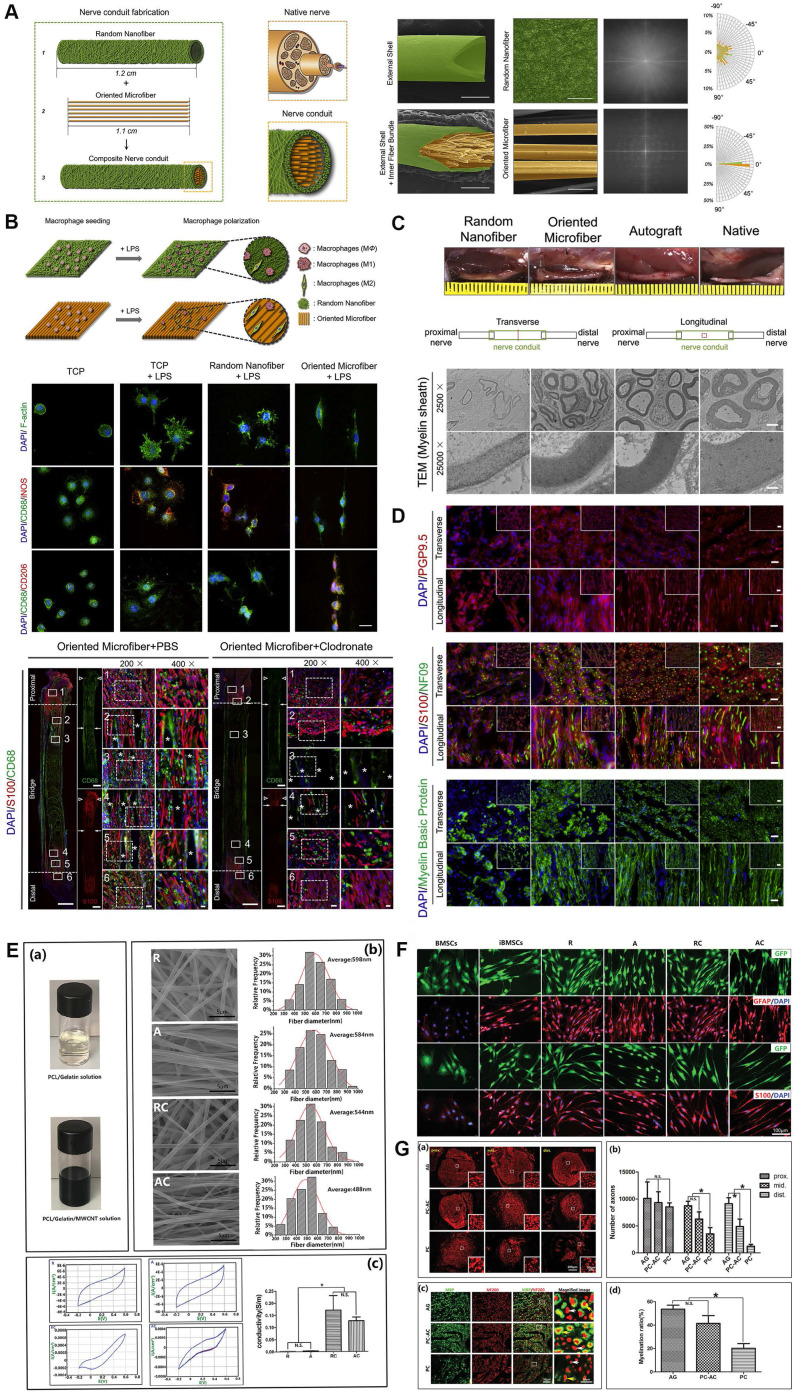
**Electrospun microfiber-fabricated nerve guidance conduits with longitudinally aligned architecture. (A)** Schematic illustration of biomimetic nerve conduit structure and the microscopic visualization of external shells and inner fiber bundle with corresponding Fourier transform images for each substrate. **(B)** Schematic illustration of the topographical effect of substrates on macrophage polarization (upper panel). Macrophage polarization in different groups was characterized by immunofluorescence staining for CD68 (M0), iNOS (M1) and CD206 (M2) (middle panel). Representative immunofluorescent images of the longitudinal sections in groups with or without macrophages at 2 weeks post-implantation (lower panel). **(C)** Macroscopic photos and transmission electron microscopic images of transverse sections of explanted conduits after 3-month implantation. **(D)** Immunofluorescent staining of the cross-sections of explanted conduits after 3-month implantation showed the distribution of pan-neuronal cells (PGP9.5), neurofilaments (NF09), Schwann cells (S100) and myelin sheath (MBP). **(E)** Digital photo of the electrospun solution, and representative SEM images of four different types of electrospun fibers, as well as recorded curves and quantitative assessments of conductivity. **(F)** Immunofluorescence micrographs of the induced cells on different nanofibers. **(G)** Immunostaining for NF200 and quantitative analysis on the transverse sections showed the regenerated axons in the proximal, middle, and distal segments of the grafts (upper panel). Double immunostaining for MBP and NF200 and quantitative analysis in the middle segments of the grafts (lower panel). Abbreviations: iNOS, inducible nitric oxide synthase; MBP, myelin basic protein; SEM: scanning electron microscope. Adapted with permission from 19, copyright 2021 Elsevier. Adapted with permission from 154, copyright 2020 Wiley.

Biomimetic nerve grafts are expected to simulate natural microenvironmental features of nervous development and repair. Currently, specialized designs aiming to mimic it are employed to modify nerve grafts, in which 2D orientational modifications on the interface are commonly endowed to the inner surface, and 3D topological designs are rendered to implants across various layers. NGCs with aligned designs on the inner surface fully conform to biomimetic rules. The aligned structures of nerve fibers as a type of topological cue would potentially promote the cell migration and rearrangement accelerating axonal regeneration 125, 148 (Figure 4). Therefore, aligned fibers and oriented micro-grooves or micro-pores, inspired by the inherent structures of nerve, are widely applied in biomimetic NGCs 149. Given the similarity between aligned architectures and nerve fibers, aligned designs are employed to create the inner walls of NGCs.

Electrospun nanofiber-manufactured NGCs, especially those with aligned nanofibers, are very promising tissue engineering strategies for injured peripheral nerve regrowth, in which all aspects of fiber diameter, porosity, and mechanical strength can be modulated by solution concentration and viscosity, temperature, humidity, and the motion of the grounded target 150. Typically, aligned electrospun PLLA nanofibers coated with decellularized peripheral nerve matrix were designed and applied in a dorsal root ganglion cultural model, and then much faster axonal extension and remyelination was observed under the topological guidance from the aligned electrospun fibers 151. In those *in vivo* studies, electrospun poly (3-hydroxybutyrate-co-3-hydroxyvalerate) (PHBV)-magnesium oleate-N-acetyl-cysteine nanofiber guidance cues and aligned poly (L-lactic acid-co-e-caprolactone) nanofibers were fabricated and employed to repair a rat sciatic nerve defect model with significantly predominant nerve fiber regeneration outcomes 20, 152. Several advanced individualized designs such as nanofiber polymers with shape memory were also used for multichannel conduit manufacture and could provide lining cues for Schwann cell migration inside 153. Interestingly, recent studies from multiple teams showed aligned nanofiber composited NGCs may facilitate electroconductivity of nerve fibers during PNR 135, 154, which was not well recognized yet and required to verify in the future.

Up to now, microfibers have been widely identified to facilitate injured neural tissue repair due to their comparable diameters to cellular units, which induced cell attachment and oriented expansion. A few techniques including dry- or wet-spinning and microfluidics are often employed to achieve their biological effects in the area of PNR. Recently, Dong *et al*. 19 designed a hollow nerve conduit enclosing aligned microfiber with a mean diameter of 27 μm by dry-spinning devices, which is appropriately suitable for cell interaction and cell-to-substrate attachment. Based on it, aligned patterns on the inner surfaces induced more macrophages recruitment and polarization to the M2 phenotype at the early stage of PNR, subsequently guiding Schwann cells and axons inside the axial track in a paracrine manner. Microfluidics-produced collagen microfibers exhibited better mechanical properties exceeding the stability of previous wet-spun collagen fibers as well as even natural tissue without the chemical cross-linking 125. However, the superiorities of nano-grade or micro-grade aligned structures, as far as mechanisms of tissue repair are concerned, remain unclear.

In addition to those axially oriented fibers, a substantial number of two-dimensional micropattern designs, such as ridge/groove arrangement, were applied to repair PNI for efficient nerve fiber extension guidance 93, 146, 147, 149. The laser-based technique 155, lithography-based technique 156 and piezoelectric inkjet printing technique 157 have been employed to fabricate oriented micropattern architectures and achieve satisfactory outcomes in cooperation with additional chemical methods. However, part of micropatterning methods such as UV light and laser tended to result in an unneglectable issue that active proteins denatured. Furthermore, it is difficult to form a perfect 3D micropattern structure, which would restrict their application in nerve tissue engineering. Therefore, a combination of multiple aligned structures above by a one-step procedure and simultaneous improvement of their limitations may lead to successful clinical translation of nerve graft products 148, 158, 159.

### 3D topological designs

**Figure 5:**
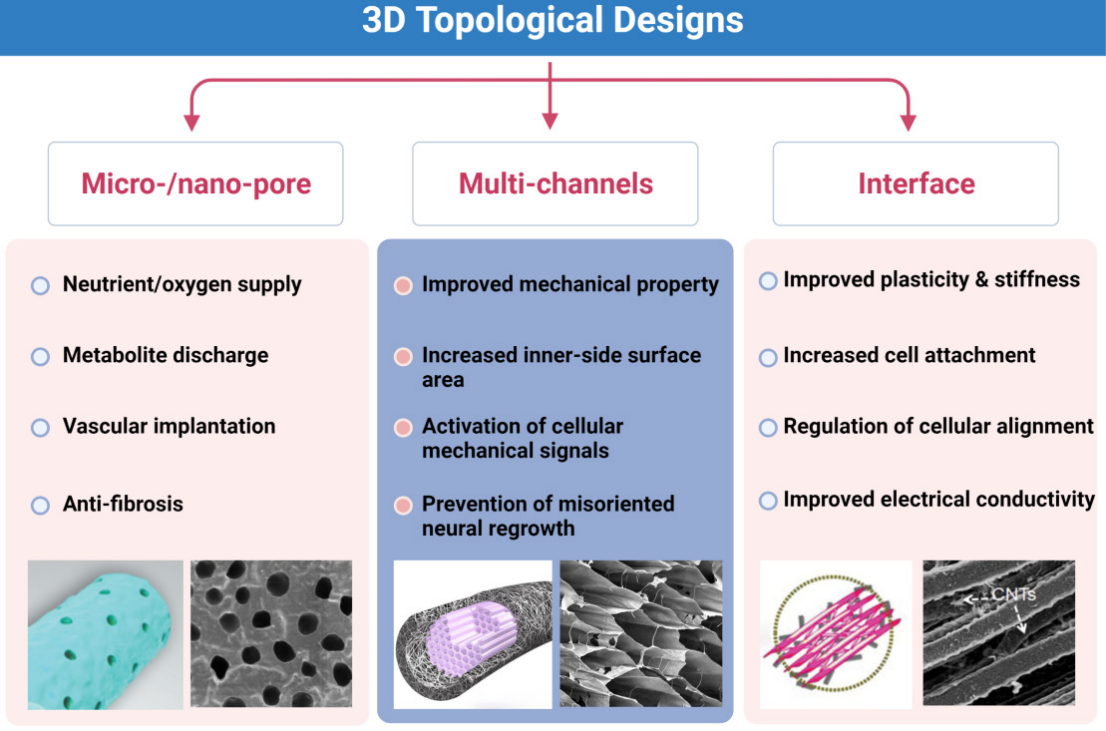
**Key points of three-dimensional (3D) topological designs.** Micro-/nano-pores on the outer surface, multi-channels and interface modifications for better outcomes of neural repair. Abbreviation: CNTs, carbon nanotubes. Adapted with permission from 135, copyright 2021 Elsevier. Adapted with permission from 162, copyright 2018 Elsevier.

As for NGCs, the key points of topological architecture include designs on the outer surface, microstructures in the lumen, and appropriate modifications of the inner layer for cell attachment (Figure 5). These modifications are expected to not only adapt to the natural physical structure of nerve fibers and rules of axonal extension but also guide mechanical signal-induced injured neural repair. First, micro-sized and nano-sized sheet-like structures in the surface are often employed to ensure permeability for nutrient and oxygen supply and facilitate vascular endothelial cell migration into injured sites 160. A few designs conforming to it demonstrated that topological architectures attenuate regional inflammatory responses, and it permits the exchange of nutritious substances and metabolites 93, 101, 160, 161. In addition, differential densities and sizes of porous structures may also contribute to the mechanical property regulation of entire scaffolds 162. However, the direct evidence that specialized topological designs in the surface modulate mechanical signals is bare.

**Figure 6:**
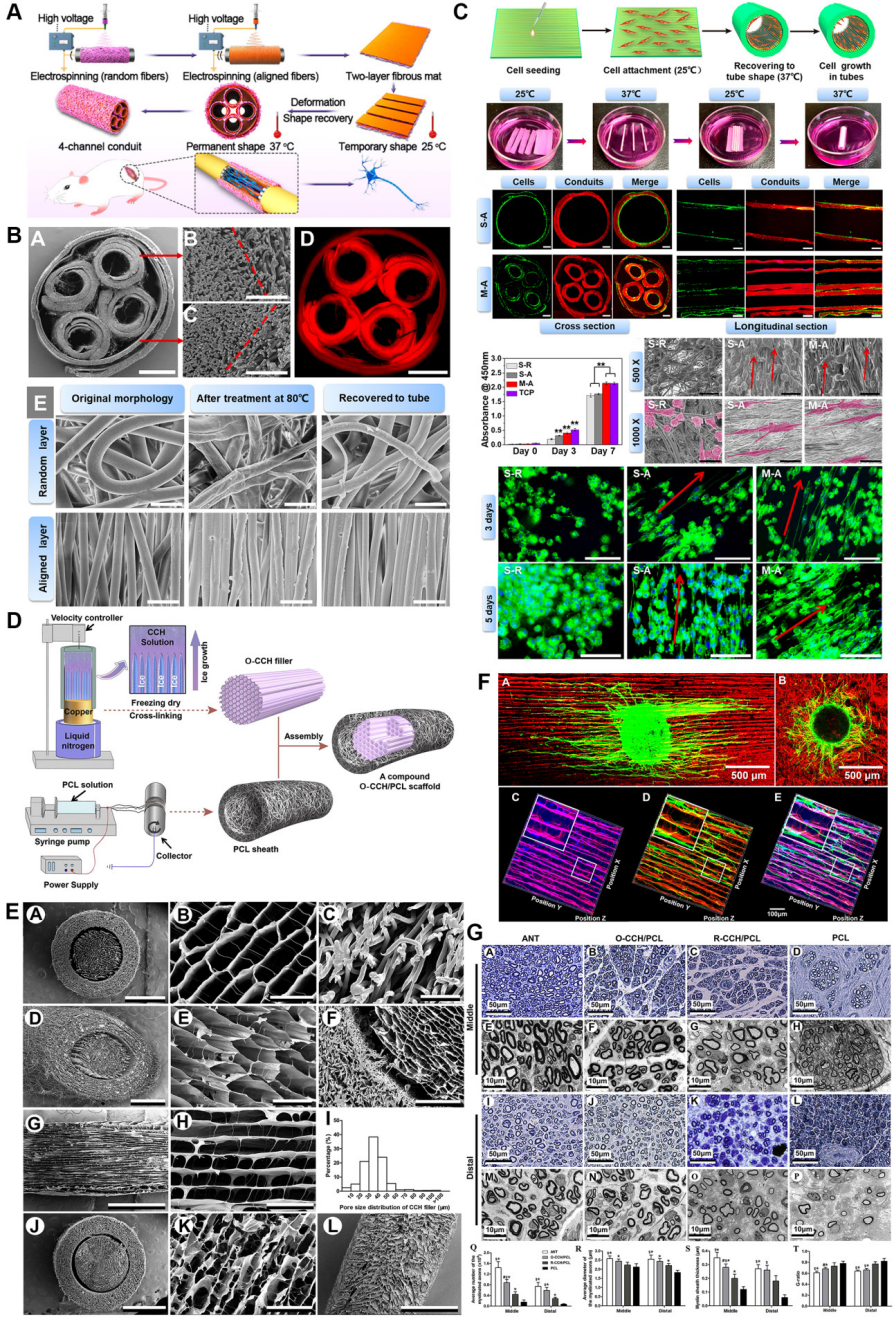
**Three-dimensional (3D) multichannel designs for peripheral nerve repair. (A)** Fabrication flow of bioinspired shape memory multichannel nerve guidance conduit. **(B)** Morphology characterizations of the fabricated multichannel nerve conduit observed by SEM and fluorescence microscope. **(C)** Schwann cell behaviors when seeding on different conduits. **(D)** Schematic illustration of the stage-wise fabrication of CCH/PCL scaffolds with uniform longitudinally oriented micro-channels. **(E)** SEM characterization of the CCH/PCL scaffold. **(F)** Morphology of DRG explants seeding on oriented or random CCH; axonal regeneration and Schwann cell migration on the CCH micro-structured filler. **(G)** Morphological appearance and morphometric assessments of regenerated nerves in the middle and distal nerve segments 12 weeks after surgery. Abbreviations: SEM, scanning electron microscope; CCH, collagen/chitosan; PCL, polycaprolactone; DRG, dorsal root ganglion. Adapted with permission from 153, copyright 2020 American Chemical Society. Adapted with permission from 162, copyright 2018 Elsevier.

The primary purposes of luminal micro-designs are always considered to increase the surface area of the inner side and improve the mechanical characteristics of scaffolds 163, 164 (Figure 6). The former point means better developing Schwann cells or immune cells attachment to a large extent for mechanosensitive receptor and channel activation, while the latter one refers to more solid architecture of multi-luminal composition than single-channel conduit. Aligned channeled structures on scaffolds physically guide terminal stumps to regrowth 153. Currently, these unique structures applied to PNR are fabricated by electrospun, directional freezing, bidirectional freezing and radial freeze-casting through temperature control, electric field or magnetic field. The physical characteristics of solutions, the molecular weight of polymers, and freezing temperatures altogether affect the direction, morphology, quantity and distribution of the porous or channeled structures 144, 165, 166. Huang *et al*. produced a compound scaffold with uniform longitudinal channeled collagen/chitosan filler by directional freezing and a porous PCL sheath, both of which were assembled for efficient peripheral nerve repair 162. In a recent study, 3D scaffolds along longitudinally oriented microchannels were fabricated by combining the modified 3D printing and directional freezing techniques, representing a promising alternative for nervous system injuries management 167.

Interface disposal plays a vital role in neural regeneration due to its direct physical touch with injured or repaired tissue in the microenvironment. In addition to the two-dimensional aligned architectures described above, the biomechanical and biophysical properties of interfaces such as plasticity and hydrophilicity modulate the cell affinity to the basal matrix 168. The interaction outcomes between cells and interfaces rest with neural outgrowth-associated behaviors of membrane proteins and cytoskeleton, and currently, both hydrophilic protein modification and hydrophobic nanoparticle immobilization are used for improving the mechanical characteristics of scaffold interface 169-171.

### Piezoelectric scaffold

**Figure 7:**
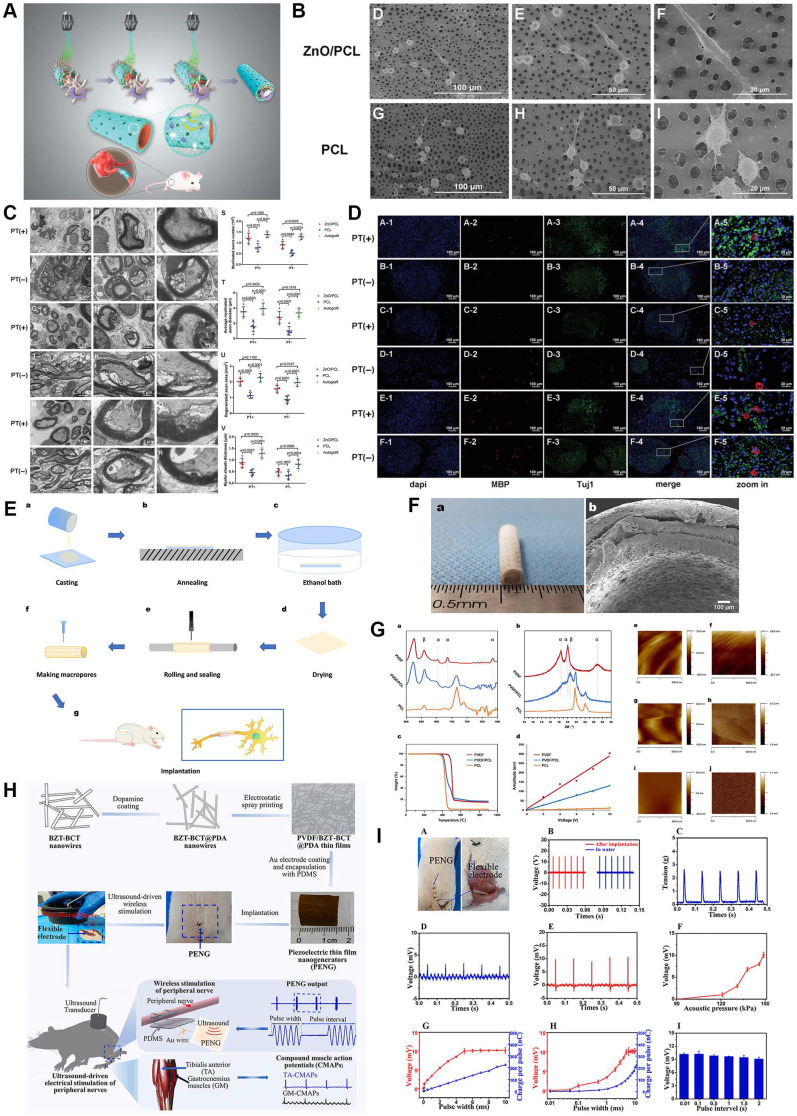
**Piezoelectric Scaffolds for neuronal regeneration and recovery. (A)** Scheme of 3D injectable multilayer fabrication for the ZnO-loaded PCL conduit. **(B)** Schwann cell morphology on the ZnO/PCL and PCL scaffolds. **(C)** Ultrathin transversal section morphology of sciatic nerves from the ZnO/PCL, PCL, and autograft groups at 18 weeks postoperatively. **(D)** Immunostaining of sciatic nerves from the ZnO/PCL, PCL, and autograft groups at 18 weeks postoperatively. **(E)** Schematic illustration of the fabrication of PVDF/PCL nerve guidance conduits. **(F)** Optical and microscopic morphology of PVDF/PCL and PCL scaffolds. **(G)** Crystalline phase analysis and piezoelectric property characterization of the electroactive scaffolds. **(H)** Illustration of the fabrication processes of PVDF/BZT-BCT@PDA piezoelectric thin film nanogenerators and the wireless electrical stimulation of peripheral nerves in rats with implantable soft piezoelectric thin film nanogenerators. **(I)** Ultrasound-driven wireless electrical stimulation of sciatic nerves based on the implanted PVDF/BZT-BCT@PDA (50%) thin film nanogenerators. Abbreviations: ZnO, zinc oxide; PCL, polycaprolactone; PVDF, polyvinylidene fluoride; BZT-BCT, 0.5Ba(Zr_0.2_Ti_0.8_)O_3_-0.5(Ba_0.7_Ca_0.3_)TiO_3_; PDA, dopamine. Adapted with permission from 18, copyright 2020 Wiley. Adapted with permission from 21, copyright 2020 Elsevier. Adapted with permission from 175, copyright 2021 Elsevier.

Biomechanical stimulation derived from the microenvironment including muscle contraction could trigger electrical activities through the application of piezoelectric biomaterials, which undoubtedly revolutionized mechanics-based tissue engineering strategies 172, 173. Moreover, intermittent electrical stimulation induced by mechanics has been identified as a positive factor during electroactive neural healing due to the electrically conductive nature of the nerve fiber. Mechanosensitive materials PLLA, ZnO, boron nitride and polyvinylidene fluoride (PVDF) have successfully transferred mechanical information they received to electron motion 18, 21, 171, 172 (Figure 7). For instance, we previously manufactured a piezoelectric zinc oxide nanogenerator scaffold for enhancing motor recovery and neural function, and implantation of zinc oxide/PCL incorporated treadmill practice provided biomimetic microenvironment and promoted nerve regeneration 18. A similar design was also applied in the area of articular cartilage repair through PLLA nanofiber-induced piezoelectric effect 174, further supporting the wide *in vivo* application of piezoelectric scaffolds. In addition, the study carried out by Chen *et al*. 175 demonstrated ultrasound-driven piezoelectric scaffolds functioned well in a segmental neural defect model through remote ultrasound-mediated strain of implanted nanogenerators. However, both extra physical exercise and ultrasound-driven strain are dependent on external force exposure, and thus safer and more convenient means should be further explored. In turn, updated research demonstrated that a flexible dielectric elastomer membrane transformed the current signal into mechanical energy 176; therefore, mechanics-electricity conversion is bidirectional by means of specially designed biomaterials.

Inherent physiological activity-mediated mechanical stress could automatically trigger the discharge on the surface of piezoelectric material 177. Cheng *et al*. 21 fabricated a porous PVDF/PCL piezoelectric composite, the electromechanical interactions of which stimulated Schwann cell proliferation and maturation, as well as morphological and functional nerve restoration. During this process, physical deformation of epineurium and other connective tissue as mechanical force sources render mechanosensitive biomaterials to charge, and then directional current converged from intermittently produced electron activities stimulates injured nerve stumps and electrically bridges them. Although only a little number of piezoelectric materials were applied in the area of PNR, there is no doubt that they are promising strategies with outstanding biocompatibility, biodegradation, mechanical properties and electrical conduction. Of note, since the majority of peripheral nerve trunks are surrounded by rich muscle mass, the invention of electrical stimulation device powered by muscular mechanical stretching is preferable and promising.

## Current knowledge gaps and challenges

**Figure 8:**
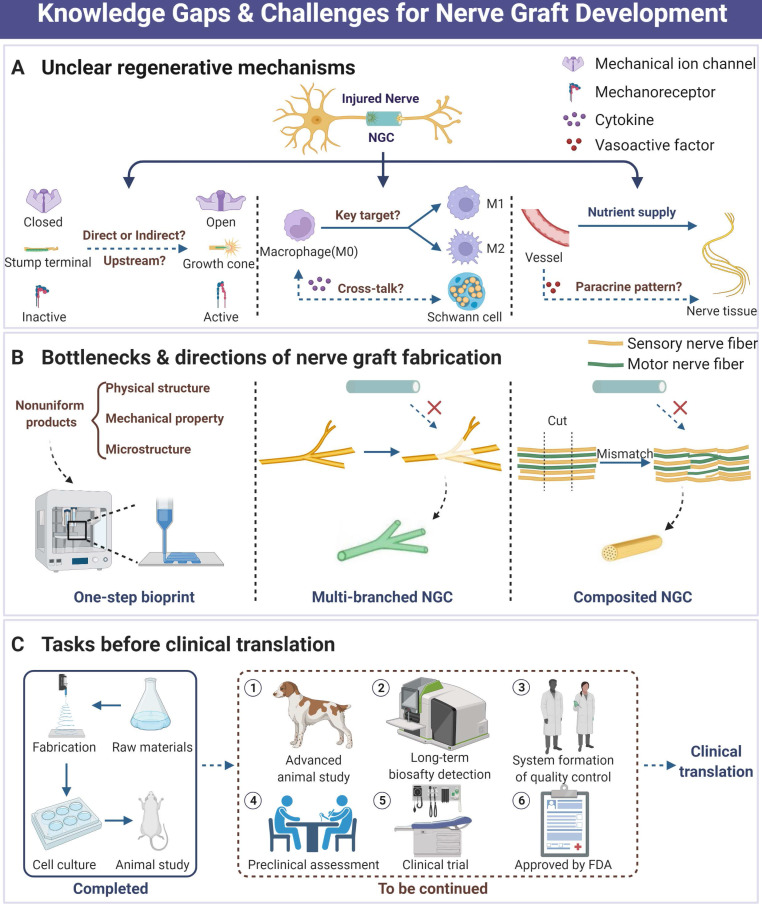
**Current knowledge gaps, practical challenges and future directions in the area of peripheral nerve regeneration. (A)** Those unclear biological mechanisms during NGC-induced nerve tissue regeneration. **(B)** The current bottlenecks of tissue engineering strategy development for nerve graft fabrication include unperfect fabrication techniques, reparative difficulties of multi-branch nerve defect, and tricky mismatch. **(C)** Necessary tasks before successful clinical translation of nerve graft products. Abbreviations: NGC, nerve guidance conduit.

To the best of our knowledge, the therapeutic strategy exploration of PNI has already been around for a long period. As nothing clinically available is perfect yet, it attracts wide attention both preclinically and clinically, especially in the area of tissue engineering and biomaterials development 17-19, 143. Based on advanced investigation for the biomechanical microenvironment of peripheral nerve, part of NGCs and other designs have exhibited comparable outcomes to autograft in animal and preclinical studies with unneglectable limitations. Currently, the following points may pose a threat to the development and application of perfect nerve grafts and deserve to be further explored (Figure 8).

The full landscape of mechanosensitive protein functions and mechanical signal transduction patterns remains to be elusive. Although a series of molecules are identified as mechanical sensors or regulators, such as Piezo1/2 and YAP/TAZ, their downstream transducers and sophisticated function in case of activation still need to be deeply investigated for better recognition 12, 24, 70, 76, 178. Moreover, the effects of physical microenvironment and substantial mechanical signals on PNR are still poorly understood, despite several studies that have observed the important significance of biomechanical microenvironment intervention 13, 19. In addition, the exploration of biological mechanisms for biomaterials-induced better nerve regeneration is rare. For instance, peripheral nerve crush or defect would be followed by the systematic immune responses, ECM structure remodeling and stem/progenitor cell recruitment 8, 81, 90, 105, 179, where macrophage polarization under the guidance of longitudinally oriented microfiber in acute stage induces the mature Schwann cell rearrangement and axonal extension with obscure spatiotemporal order 19. Neovascularization is the prerequisite of tissue regeneration, and endothelial progenitor cell mobilization not only directly participates in vessel formation but also plays a vital role in microenvironment modulation in a paracrine manner 180, 181. However, fewer previous studies about topological designs of NGCs involved in the landscape of neovascularization regulation and subsequent paracrine factor-induced nerve repair. Despite fewer reports involved in the investigation of lymphatic system destruction and reconstruction accompanied by PNR, their potential effects on microenvironmental homeostasis maintenance are indispensable due to the necessities of lymphatic circulation and robust immunomodulatory function. Much clearer mechanisms will undoubtedly facilitate refined biomaterials design and application.

A few details about nerve graft fabrication should be taken seriously. First, the limitations of bilayer and multilayer nerve grafts, especially compatibility of interface restrict their application scene. Numerous traditional NGCs are designed with inner hydrophilic surfaces for regeneration and outer hydrophobic characterization for anti-fibrosis 168, 182, while a majority of interface disposals among layers are commonly neglected, leading to poor stability of nerve graft products. To address this issue, one-step fabrication employing advanced techniques including additive manufacturing may be preferable and tend to produce perfect grafts 183. In addition, since nerve graft with multiple branching is quite difficult to accurately design and fabricate, the nerve fiber injuries often fail to manage and cure in clinical practices if autograft is unavailable when multiple branching nerve suffering critical defect 184-186. Therefore, the concept of “precision medicine” should be rendered into PNR and radiological methods such as ultrasound and digital fluorescent reconstruction will be employed to acquire the parameters of injured nerves, ensuring appropriate graft customization. Due to the different diameters and distribution of fascicle-like structures within different nerves (e.g. sciatic nerve and sural nerve), the optimal parameters of intraluminal modifications vary in different situations and are worthy of further exploration. Another tricky problem is that heterogeneous nerve fiber mismatch during PNR always poses a threat to sensory and motor function recovery 145, 187, 188. As previous studies have pointed out that physical features of biomaterials surface were associated with cell fate decision and various activities 189, future work is needed to explore structural and biomechanical preferences in the process of sensory or motor nerve fiber repair. Thus, elaborate specialized designs for each type of nerve fiber will be assembled into one compound graft and avoid fiber mismatch in the future. The main task in future decades is how to harness the biomechanical characterizations for PNR. Primarily, the mechanical preferences and optimized substrate stiffness of sensory, motor or autonomic nerve fibers are expected to be elaborately clarified. According to these theories, specialized nerve graft designs and materials for meeting mechanical requirements will serve as common strategies, and thus “precision management” will be well achieved. In addition, although orientation architectures are often employed to fabricate nerve graft, the intracellular cascades induced by mechanoregulatory elements remains poorly understood when cell attachment to aligned substrate. This issue proposed an enormous research area that how microstructures of substrate affect cell activities, which will help to modulate the shape and size of microstructures once clear identification. Finally, piezoelectric graft and self-powered device by means of mechanics-electricity transformation deserve to be deeply investigated, in which appropriate electrode design is the key to translational application.

A few issues about biosafety and biocompatibility of biomaterials should be well addressed before clinical translation. Despite a wide range of nerve grafts displaying satisfactory regenerative outcomes, fewer of their additives are FDA approved, posing a major threat to biosafety and biodegradebility 190, 191. Toxicity tests in their research are often carried out in a short time, while in clinical practice nerve graft application is undoubtedly long-term. This contradictory point is primarily attributed to the potential harm (e.g. toxicity and immunogenicity) of active or non-active degradation products, as well as obscure mechanisms. On the other hand, variations of component contents and environmental conditions sensitively affect mechanical properties and microstructures of products such as porosity, which will fail to meet clinically practical requirements that medical materials should be highly uniform. Based on these concerns, biomimetic compositions using simple or less basal materials such as PCL by means of topological modifications and mechanical property modulations may be superior to biological or chemical additives for clinical product development. Finally, there are fewer standards of quality control for clinical peripheral nerve grafts. For example, neither evaluation methods nor standard guidelines of various parameters are well established, but it is an issue that must be resolved to achieve clinical translation.

## Summaries and prospects

The biomechanical microenvironment always modulates the precise activities of various cell types and non-cellular structures in peripheral nervous system from embryonic development to physiological signal transduction after birth, altogether maintaining its nutritional balances and biological functions. The striking regenerative potential of the peripheral nervous system is also triggered by local physical structures and biomechanical signals from ECM, immune cells and neural cell populations in case of PNI. Thus, a wide range of tissue engineering strategies with two-/three-dimensional unique topological designs continuously emerge, adapting to biomechanical microenvironment-induced nerve fiber repair. With the progress of underlying mechanism investigation and advanced concept introduction, the bottleneck of therapeutic means will be surmounted, whereas those novel tissue engineering strategies with refined designs and techniques tend to achieve satisfactory outcomes, paving the way for preclinical research and even clinical translation.

## Figures and Tables

**Table 1:** Mechanical properties of tissue engineering nerve grafts

Materials	Fabrication technique	Fiber diameter (μm)	Elastic modulus (MPa)	Elongation at break (%)	Microstructure	Reference
PCL, graphene	3D fabrication	/	68.7	/	porous network	17
PCL	electrospun	3.10 to 4.60	19.4	5.2	axial orientation	129
PCL, nanodiamond	3D spraying	/	72.9	48.7	microporous dotting	133
PCL, graphene	chemical vapor deposition	/	2.67	/	porous network	130
PCL, ZnO	3D spraying	/	68.46	47.5	microporous structure	18
PCL, Fe_3_O_4_-MNPs, melatonin	electrospun	0.12	2.58	75.9	microhole, porous network	134
PCL, carbon nanotubes	electrospun	4.15	65.6	/	aligned structure	135
collagen type I	microfluidics	3.69	4138	25	axial orientation	125
collagen type I, chondroitin-6-sulfate	snap cooling, freeze drying	/	/	/	aligned microporous architecture	136
collagen, PLCL	3D bioprinting, electrospun	0.65	/	/	aligned structure	131
PLCL, PDS	electrospun, melt spun	27.10	/	/	axial orientation	19
chitosan, peptides	electrospun	8 to 10	3.1 kPa	39.5	aligned structure	137
PLLA	extrusion	/	/	/	porous architecture	138
PLLA, dnECM	electrospun	0.65	/	/	aligned structure	139
PLLA, PLCL	electrospun	0.60	/	/	aligned structure	140
PLLA, soy protein	electrospun	0.35 to 0.98	1.99 to 3.47	3.44 to 26.65	aligned structure	141

**Abbreviations**: PCL, polycaprolactone; PLLA, poly (L-lactic acid); PLCL, poly (L-lactide-co-ε-caprolactone); PDS, polydioxanone; ZnO, zinc oxide; MNPs, magnetic nanoparticles; dnECM, decellularized nerve extracellular matrix.

## Abbreviations

PNI: peripheral nerve injury
PNR: peripheral nerve regeneration
FAK: focal adhesion kinase
YAP: yes-associated protein
TAZ: transcriptional coactivator with PDZ-binding motif
ECM: extracellular matrix
sCAMs: synaptic cell adhesion molecules
CAMs: cell adhesion molecules
MRTFs: myocardin-related transcription factors
SRF: serum response factor
TRP: transient receptor potential
NMDA: N-methyl-D-aspartate
TRAAK: TWIK-related arachidonic acid-activated K^+^
AHTMR: fast-conducting myelinated high-threshold mechanoreceptors
HCN: hyperpolarization-activated cyclic nucleotide-gated cation
NGC: nerve guidance conduit
2D: two-dimensional
3D: three dimensional
FDA: Food & Drug Administration
PCL: polycaprolactone
L-lactic acid): PLLA, poly
PLCL: poly (L-lactide-co-ε-caprolactone)
PDS: polydioxanone
ZnO: zinc oxide
MNPs: magnetic nanoparticles
dnECM: decellularized nerve extracellular matrix
PHBV: poly (3-hydroxybutyrate-co-3-hydroxyvalerate)
PVDF: polyvinylidene fluoride
